# The Atlanta Society of Medicine

**Published:** 1890-04

**Authors:** 


### Society Notes.

### THE ATLANTA SOCIETY OF MEDICINE.

March 18, a regular meeting was held with Dr. Cooper in the chair. Dr. Gaston reported a case of urinary calculi that he saw in consultation with Dr. Horsley, of West Point. Crushing had been tried but without success, so that the catheter had to be used frequently. Finding an enlarged prostate gland the supra-pubic operation was selected as offering the best chance. The bladder was filled with water and a Barnes dilator put in the rectum; in this way the bladder was made quite prominent. The incision was made in the median line, and the tissue carefully cut through, the bladder was exposed, when it was caught with a tenaculum, needles armed with four strands of silk were passed, two stitches being taken on each side, and the bladder thus secured to the skin. The bladder was then opened and twenty-eight calculi of various sizes were washed and picked out.

The prostate gland was so enlarged and projected so much into the bladder that it formed large pouch behind it, thus accounting for the failure in attempting to crush the stones.

Two drainage tubes were fastened in and to the drainage tubes were attached condomes to catch the urine.

Dr. Elkin states that he was well pleased with the operation and did not think that the danger from wounding the Peritoneum was as great as is supposed.

Dr. Huzza presented two ovaries that he had just removed and stated that he would give a full report of the case at some future time.

Dr. Stockton reported a case of fish bone in the larnynx. It was lodged between the true and false cords in such a position that a probang could not catch it, several physicians having tried. By the use of the laryngoscope it was removed without trouble at the first attempt.

Dr. Gaston reported a case of pin lodged in the Pharnynx, when he had a good deal of trouble in getting it out as both head and point were ingaged in the tissues.

Dr. Noble reported a case of the prolapse of both ovaries in which the patient suffered from a sort of epileptic attacks.

Dr. Hardon presented a case of superficial papillomata from a patient 62 years old.

The society then held an informal discussion on skin diseases in general and acne in particular.

